# A set of highly informative rat simple sequence length polymorphism (SSLP) markers and genetically defined rat strains

**DOI:** 10.1186/1471-2156-7-19

**Published:** 2006-04-04

**Authors:** Tomoji Mashimo, Birger Voigt, Toshiko Tsurumi, Kuniko Naoi, Satoshi Nakanishi, Ken-ichi Yamasaki, Takashi Kuramoto, Tadao Serikawa

**Affiliations:** 1 Institute of Laboratory Animals, Graduate School of Medicine, Kyoto University, Kyoto 606–8501, Japan

## Abstract

**Background:**

The National Bio Resource Project for the Rat in Japan (NBRP-Rat) is focusing on collecting, preserving and distributing various rat strains, including spontaneous mutant, transgenic, congenic, and recombinant inbred (RI) strains. To evaluate their value as models of human diseases, we are characterizing them using 109 phenotypic parameters, such as clinical measurements, internal anatomy, metabolic parameters, and behavioral tests, as part of the Rat Phenome Project. Here, we report on a set of 357 simple sequence length polymorphism (SSLP) markers and 122 rat strains, which were genotyped by the marker set.

**Results:**

The SSLP markers were selected according to their distribution patterns throughout the whole rat genome with an average spacing of 7.59 Mb. The average number of informative markers between all possible pairs of strains was 259 (72.5% of 357 markers), showing their high degree of polymorphism. From the genetic profile of these rat inbred strains, we constructed a rat family tree to clarify their genetic background.

**Conclusion:**

These highly informative SSLP markers as well as genetically and phenotypically defined rat strains are useful for designing experiments for quantitative trait loci (QTL) analysis and to choose strategies for developing new genetic resources. The data and resources are freely available at the NBRP-Rat web site [1].

## Background

Over the last several years, rat genomic data has grown dramatically, including the genomic sequence [2], single nucleotide polymorphisms (SNPs) [3], and microarray gene-expression profiles [4]. Several rat genetic markers, such as simple sequence length polymorphisms (SSLPs), expressed sequence tags (ESTs) and sequence-tagged sites (STSs), have also been developed and integrated into a high-density rat radiation hybrid map [5], resulting in the comparative mapping of different species, such as between humans and mice. Such infrastructure development of rat genomic resources provides important information for improving the functional annotation of genomic sequences underlying rat QTL and identifying candidate genes for human complex diseases. Although more than 1000 quantitative trait loci have been mapped thus far onto rat chromosomal regions [6], only a few of the mapped QTLs have been identified at the molecular level, such as those for type-I [7] and type-II [8] diabetes, arthritis [9], or fatty acid metabolism [10]. There is a need for functional infrastructure improvement not only for genomic resources and experimental tools, but also for animal resources themselves.

The National Bio Resource Project for the Rat (NBRP-Rat) was started in July, 2002 [11]. The major aim of this project is to improve the experimental environment of rat research by collecting existing rat strains, cryopreserving their sperm and embryos, and distributing them to interested researchers. Until now, more than 300 rat strains have been deposited into the NBRP-Rat. They are all indexed in a publicly accessible database [12]. The biological resource at NBRP-Rat provides the opportunity to supply any deposited rat strain on request to interested scientists around the world, allowing researchers to accelerate their research pace, and to conserve animals and money. Furthermore, to enhance the value of the collected strains and to supply well-characterized rats to the research community, we are promoting the Rat Phenome Project [13], which describes many deposited rat strains with wide-ranging phenotypic measurements comprising 109 parameters: functional observational battery (neurobehavior), behavioral studies, blood pressure, biochemical blood tests, and hematological, urological, and anatomical parameters. These data can be systematically viewed by 'strain ranking' for each parameter, which allows researchers to easily and simultaneously compare phenotypic values of multiple strains and to identify new rat models for specific experiments.

In parallel with the Rat Phenome Project, we are genotyping deposited rat strains. The two major objectives of this study were (1) to choose highly polymorphic SSLP markers that would facilitate genome-wide scans in as many as possible crosses between inbred strains of rat, and (2) using these markers to genetically evaluate rat strains deposited into NBRP-Rat. These highly informative SSLP markers in combination with phenotypically defined rat strains are powerful tools for researchers to design various experiments, such as for assessing QTL phenotypes.

## Results and discussion

### Selection of rat SSLP markers

**Figure 1:**
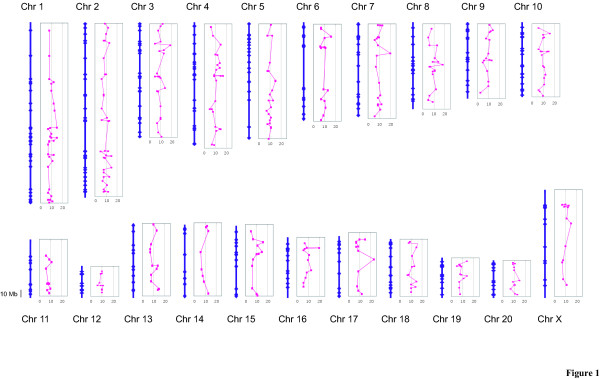
**A physical map of 357 rat SSLP markers. **The blue lines represent the chromosomal physical map derived from the Rat Genome Browser [15][16] and the blue dots represent the physical position of SSLP markers along the chromosome. The right side graphs of the chromosome indicate the number of alleles among genotyped 122 strains as red dots.

A panel of 384 SSLP markers was first selected from publicly available data in the Rat Genome Database [14] with two objectives: (1) to obtain the maximum polymorphisms among the listed rat inbred strains and (2) to cover the rat genome except for chromosome Y. Out of 384 markers, 18 failed to be or were poorly amplified by PCR, 7 were amplified as double or multiple bands, and 2 turned out to be designed for the same microsatellite region as other markers. A total of 357 markers were finally used for subsequent genomic screening of rat inbred strains (Fig. 1 and Supplementary Table 1), of which 323 were identified by their physical position in two genome sequence databases, the Ensembl Genome Browser [15] and UCSC Genome Browser [16]. All 34 unidentified markers of the rat genome sequence have been mapped onto a previously reported linkage map (SHRSP × BN cross) [17] or on the radiation hybrid map (RH map version 3.4) [17] published by RGD [14]. The average distance of the marker was 7.59 Mb with the largest gap of 63.0 Mb around the center region of chromosome 6, which roughly corresponds to 28 cM. Detailed information on the SSLP marker set used is available on our website [18].

**Table 1:** Polymorphic rate between 25 commonly used inbred rat strains

	ACI	BB	BN	BUF	DA	DON	F344	FH	GK	IS	KYN	LE	LEC	LEW	NER	OM	PVG	RCS	SHR	TM	W	WAG	WBN	WKY	WTC	**Total**
ACI/Nkyo	-	76	83	65	31	84	65	75	79	88	85	75	78	69	70	76	71	73	84	75	83	70	71	81	80	**74 **
BB/WorTky		-	80	66	78	83	73	68	79	87	84	77	76	60	68	74	69	67	77	72	76	60	71	77	82	**74 **
BN/SsNHsd			-	80	81	92	83	80	86	91	90	85	85	78	82	86	85	82	91	83	82	78	81	87	89	**83 **
BUF/NacJcl				-	64	86	61	71	76	89	85	75	76	64	67	73	66	65	80	66	79	62	69	77	80	**72 **
DA/Slc					-	83	64	75	77	89	85	73	74	67	70	74	68	66	83	74	81	64	74	78	76	**77 **
DON						-	84	87	62	73	73	90	90	85	84	88	85	87	57	81	78	84	86	53	55	**76 **
F344/Stm							-	72	80	89	86	73	73	66	64	69	62	64	81	72	80	61	67	77	78	**74 **
FH/HamSlc								-	79	86	85	67	69	64	77	78	76	73	84	69	81	67	74	82	80	**76 **
GK/Slc									-	79	75	83	83	76	76	82	79	79	67	76	66	73	77	60	58	**78 **
IS/Kyo										-	80	90	90	88	90	89	87	89	73	87	83	87	87	75	75	**84 **
KYN/Hok											-	86	87	86	83	87	84	85	76	86	65	81	85	75	74	**80 **
LE/Stm												-	50	73	76	72	77	73	86	68	81	72	75	83	85	**77 **
LEC/Hok													-	74	79	75	78	77	86	69	85	73	76	84	83	**76 **
LEW/SsNSlc														-	65	73	69	67	83	71	73	59	69	75	79	**76 **
NER															-	71	63	65	81	73	74	68	67	78	82	**76 **
OM/NSlc																-	69	64	82	75	78	67	73	82	84	**75 **
PVG/Seac																	-	48	83	75	77	63	67	81	83	**73 **
RCS/kyo																		-	80	73	77	53	70	78	81	**78 **
SHR/Izm																			-	79	74	83	82	50	52	**74 **
TM/Kyo																				-	78	69	74	76	77	**77 **
W/Kyo																					-	76	82	69	70	**70 **
WAG/RijKyo																						-	62	77	80	**75 **
WBN/KobSlc																							-	81	83	**76 **
WKY/Izm																								-	30	**75 **
WTC																									-	**75 **

### Genomic profiles and chart tools

We genotyped 122 rat strains with 357 SSLP markers, including 65 inbred strains, 22 substrains, 29 recombinant inbred (RI) strains, and 6 wild rats. The allele size data for each strain are available as genetic profiles on our SSLP database [18]. On the genomic profiles pages, the genotyped strains can be sorted according to their allele size for each marker to easily compare their genetic differences. Researchers can also select any pairwise combination or multiple strains to a maximum of 5 strains to retrieve informative markers for each possible cross.

**Figure 2:**
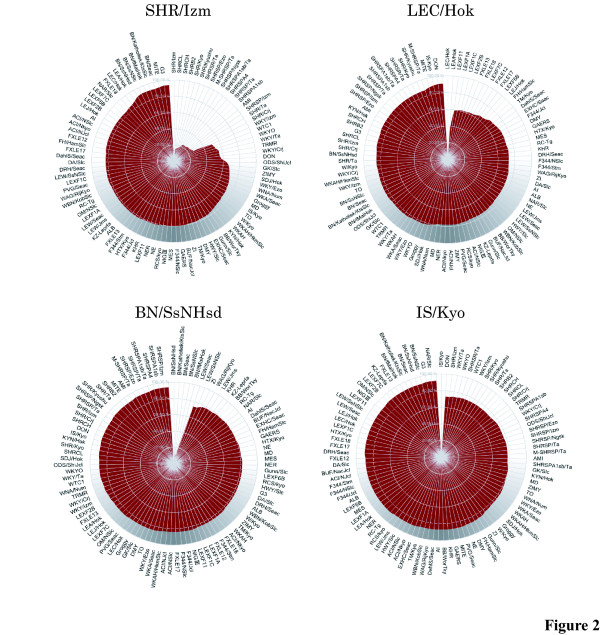
**A pedigree-like charting tool on the SSLP database. **A pedigree-like charting tool that displays SSLP differences among the genotyped rat strains on the SSLP database [18]. Researchers can select a rat strain to instantly compare its genetic background against all rat strains typed at NBRP-Rat.

In addition to the genomic profiles, we have introduced a pedigree-like charting tool that displays genetic differences among the genotyped rat strains (Fig. 2). By selecting one rat strain, its genetic background is instantly compared against all of the strains genotyped at NBRP-Rat. This allows for the selection of a crossing partner strain for QTL analysis or for the easy construction of congenic strains. For instance, BN strains would be good candidates to be crossed with SHR/Izm for genetic analysis, owing to their large genetic diversity (Fig. 2).

Single nucleotide polymorphisms (SNPs) have been assumed to be the best source of genetic variations, accounting for quantitative phenotypic differences between individual strains, and thus became the most promising genetic markers for QTL mapping on dense physical maps [19]. Recently, more than 11,000 rat SSLP markers have been constructed and are publicly available [20]. However, SSLP markers have proved and continue to be a useful genetic tool, particularly in initial mapping studies, owing to their great variety in product size, their easier detection and equal distribution on the genome. In our project, we selected 357 SSLP markers which are equally distributed through the whole rat genome for genetic screening of various rat strains (Fig. 1). The average number of marker alleles among the 122 rat inbred strains tested here was 9.78 ± 2.87, indicating high polymorphisms of the markers. This allows researchers to select informative SSLP markers throughout the rat genome to evaluate their genetic background.

### A set of genetically defined rat strains

The 122 rat strains tested here comprise 7381 potential crossing pairs. The polymorphic rate between all possible pairs is shown in supplementary Table 2. Among all crossing pairs, 19.3% have more than 300 informative markers and 87.9% have at least 200 informative markers out of our set of 357 markers (supplementary Fig. 1). The greatest number of informative markers is 341 (96%), derived from the pair between the German wild rat G3 and SHRSR/Ta. The average number of informative markers for a potential cross was 259 ± 21 SD (72.5% of 357 markers), indicating a higher polymorphic rate of our SSLP marker set than that for previously reported data [21,22].

Table 1 indicates the polymorphic rate among 25 commonly used rat inbred strains. All pairs between the strains have at least 107 polymorphic markers (more than 30%). The average number of markers for crosses between 25 commonly used laboratory strains was 271 ± 11 SD (76.0%), which corresponds to an average marker density of 10.0 Mb or 5.6 cM. The highest rate of polymorphisms was 92% between BN and DON. BN, whose genome was closely sequenced [2], has often been used as a crossing partner for linkage and QTL analysis because of its genetic diversity from other inbred rat strains. In contrast to this general assumption, our data suggest it could be promising for certain genetic studies to use the IS strain because of its higher average rate of polymorphisms and various interesting phenotypes (hypotonic, low cholesterol, etc.), compared to commonly used rat strains such as BN. Given that the IS strain was established by crossing a Japanese wild male and a Wistar female as a model for vertebral malformation [23], this strain is a useful but almost unused genetic resource.

**Figure 3:**
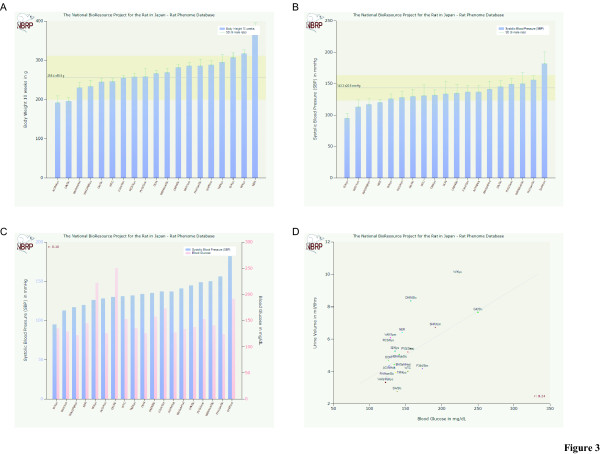
**A graphical charting tool on the Phenome database. **A graphical charting tool that displays phenotypic values for the deposited rat strains at NBRP-Rat on the Phenome database [13][24]. Various rat strains can be sorted according to their values for a selected parameter, such as body weight (A) and blood pressure (B). Column chart (C) or a scatter plot (D) are also two available options in case two parameters are selected to compare their correlation across all examined rat strains.

Phenotype data are also available for the above genetically defined rat strains on our phenome database [24]. A listing, known as 'strain ranking' (Fig. 3), allows the sorting of more than 100 rat strains according to their phenotypic values. For instance, Figures 3A and 3B indicate that commonly used inbred strains have a wide variability of values for phenotypic parameters, such as body weight and blood pressure, respectively. The charting tool can also provide a column chart or a scatter plot for two selected parameters to indicate the correlation between selected rat strains (Figs 3C and 3D). These phenotype and genotype data allow for the easy selection of appropriate rat strains for various research fields [13].

### A family tree of inbred rat strains

**Figure 4:**
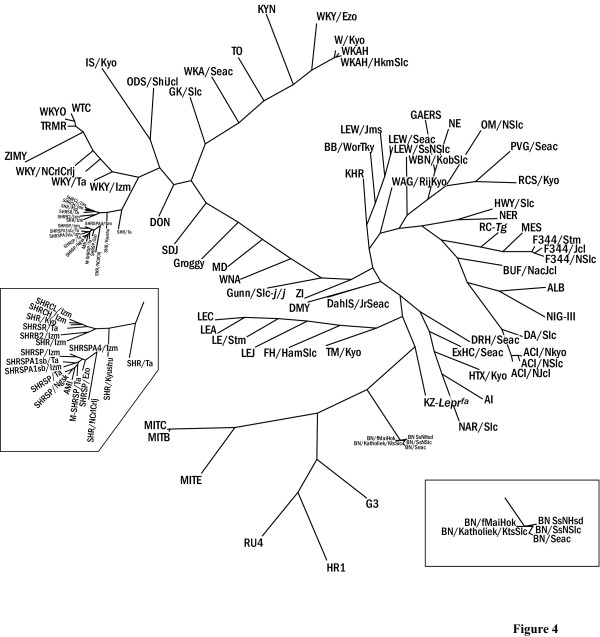
**A family tree of 93 inbred rat strains. **A phylogenetic tree was developed through a heuristic search for maximum parsimony implemented in PAUP 4.0b10 [25]. TreeView [32] was used to display the radial tree.

Information about the relationships between inbred rat strains is useful for determining the extent of polymorphisms between strains when designing crosses and for evaluating the genetic background of strains when assessing phenotypes. Using the genotype profile derived by our 357 SSLP marker set, we constructed a phylogenetic tree for 93 rat strains (Fig. 4). This tree includes all genotyped strains except for recombinant inbred (RI) ones. Although German and Japanese wild rat strains have not yet been deposited in NBRP-Rat, we used their DNA for phylogenetic analysis to extend the genetic range of our approach and to classify the genetic basis of commonly used laboratory strains. Maximum parsimony analysis was implemented through a heuristic search in PAUP 4.0b10 [25].

As shown in Fig. 3, the phylogenetic relationships of rat strains in the family tree were well resolved and appeared to be mostly consistent with their known histories [26] and previously reported rat family trees [21,22]. As expected, Japanese and German wild rats as well as BN strains, which originated from a brown mutant in a stock of wild rats maintained by DH King and P Aptekman in Philadelphia [26], were distant from each other and from other strains of commonly used laboratory rats. It is assumed that a large proportion of laboratory rat strains originated from the Wistar strain, which were brought to the Wistar Institute by Donaldson in 1906. However, the origin of this strain is still unknown [27]. In the family tree, all Wistar strains including those bred in Japan as well as in other countries, are apparently very different from the group of wild rat strains and BN strains. Further genetic analysis of wild rats derived from other countries, such as China or India, or fancy rats might clarify the origin of the laboratory rat, especially the Wistar-derived strains. In mice, the majority of laboratory strains not derived from wild species are assumed to have originated from a relatively small group of ancestral subsets [28]. In the rat family tree, the laboratory rat strain KZ-*Lepr*^*fa*^, which originated from Zucker-fatty rats, belongs to a subgroup of wild rats. Furthermore, IS and KYN strains are historically known to be established by crossing Wistar rats with Japanese wild rats [23]; [29], and this is supported by their longer branch length in our topological tree and relatively higher polymorphic rate from other laboratory strains (Table 1). These observations mean that such inbred strains of rat can provide alternative genetic variations when assessing QTL phenotypes.

## Conclusion

The most unique and important point of this study is the availability of many inbred rat strains, which have been genetically defined with a set of equally distributed SSLP markers present throughout the rat genome. Rat strains have also been systematically characterized regarding phenotypic measurements comprising 109 parameters in the Rat Phenome Project [13]. These genetically and phenotypically characterized rat strains can be freely acquired from NBRP-Rat with a contracting material transfer agreement (MTA). Researchers can easily search for their strains of interest according to their phenotypic and genetic requirements by comparing more than 100 inbred strains using visual charting tools in our Rat Phenome and SSLP database. A catalogue of these rat strains provides the opportunity to identify the most suitable parental strains for setting up an appropriate cross to identify QTLs. In addition, this unique catalogue shows the general range of phenotype and genotype data for many inbred strains.

## Methods

### Animals

All deposited rats at the NBRP-Rat are maintained in specific pathogen-free (SPF) areas or are cryopreserved. Detailed information concerning deposited strains, the depositor, origin, generations, references, deposition status, usage restrictions etc., can be obtained from our website [1]. MITB, MITC, and MITE are Japanese wild-rat strains (partially inbred) of Mitake B, C, and E [30], while HR2, G3, and RU1 are individual males of German wild rats [31]. Although these wild-rat strains and some commercial strains have not yet been deposited in NBRP-Rat, we used their DNA as controls and for genetic enrichment for our phylogenetic analysis. For all markers that appeared to be bi-allelic, in particular for the wild German rats, we always considered only the shorter allele for phylogenetic calculations.

The genomic DNA of the deposited rat strains at NBRP-Rat was extracted from the tail. The tip of the tail was digested with lysis buffer (100 mM Tris- HCl, 12.5 mM EDTA, 150 mM NaCl, 1% sodium dodecyl sulfate (SDS), 0.8 mg/ml proteinase K) at 50°C overnight. Genomic DNA was extracted using the automatic DNA purification system GENEXTRACTOR TA-100 (Takara). DNA from the German wild rats, HR2, G3, and RU1, was provided by Ingrid Klöting (University of Greifswald).

### Primers and PCR

Primers for the 384 SSLP markers tested here were selected from the Rat Genome Database [14]. Detailed information on the primer set is available on our website [18]. PCR reactions were carried out in a total volume of 25 μl under the following conditions: 94°C for 3 min for 1 cycle, 94°C for 30 sec, 60°C for 30 sec, and 72°C for 1 min for 35 cycles. The final reaction conditions were 100 ng genomic DNA, 200 μM each dNTP, 1.0 mM MgCl_2_, 0.66 μM each primer, and 0.4 U Taq DNA polymerase (GibcoBRL). The forward primer of each pair was labeled on the 5' end with a fluorescent dye. The size of the PCR products was determined with internal size standards in each capillary on an ABI3100 DNA sequencer (Applied Biosystems).

### Phylogenetic analysis

A phylogenetic tree of rat strains was obtained through maximum parsimony analysis implemented in PAUP 4.0b10 [25]. First, the allele size of the 357 SSLP markers was transformed to discrete characters in such a way that any size allele differed by one step. A heuristic search method was used in PAUP to search for optimal trees because the genotype data set for the 122 rat strains was too large for single-step computer analysis. Unordered characters were defined such that any state was capable of transforming directly to any other state with equal cost (Fitch parsimony). Two basic strategies are combined in the heuristic search strategy: a stepwise addition to obtain an initial tree, and branch swapping for rearrangement to find shorter trees. Under the analysis, 100 random addition-sequence replications were done, followed by a tree bisection-reconnection (TBR) branch swapping algorithm with the COLAPSE option on to collapse any zero-length branches and with the STEEPEST DESCENT option off. Tree stability was estimated by bootstrap analysis of 100 replicates, sampling characters with equal probability. TreeView [32] was used to display the radial tree.

## Authors' contributions

TM selected SSLP markers, performed phylogenetic analysis and wrote the manuscript. BV constructed the website, developed a pedigree-like charting tool, and assisted with the computational analysis of the results. TT contributed to the initial analysis of genotyping data. KN contributed to collecting animals. SN prepared genomic DNA and performed PCR reactions. KY contributed to breeding animals. TK participated in protocol development and results' interpretation. TS conceived, designed, and coordinated the study, and revised the manuscript. All authors read and approved the final manuscript.

## Supplementary Material

Additional File 1 **supplementary table **1**Information on 384 SSLP markers selected in this study.** Click here for file

Additional File 2 **supplementary table 2 Polymorphic rate between all possible pairs of 122 rat strains.** Click here for file

Additional File 3 **supplementary Figure **1**The number of informative markers per cross. **Pairwise combinations of 122 inbred rat strains were analyzed to determine the number of markers that were polymorphic for each cross. The number of informative crosses was plotted against the number of informative markers for each pair of strains. The tail on the left side of the histogram displays crosses between closely related substrains, including a cluster of ACI, BN, F344, and SHR strains. The mean number of informative markers per cross for all pairs of strains analyzed was 259 ± 21 SD (72.5% of 357 markers). Click here for file
