# Supplementary figures and images for: A set of highly informative rat simple sequence length polymorphism (SSLP) markers and genetically defined rat strains
Source: BMC Genet. 2006 Apr 4;7:19. doi: 10.1186/1471-2156-7-19 (PMC1475628; doi:10.1186/1471-2156-7-19)

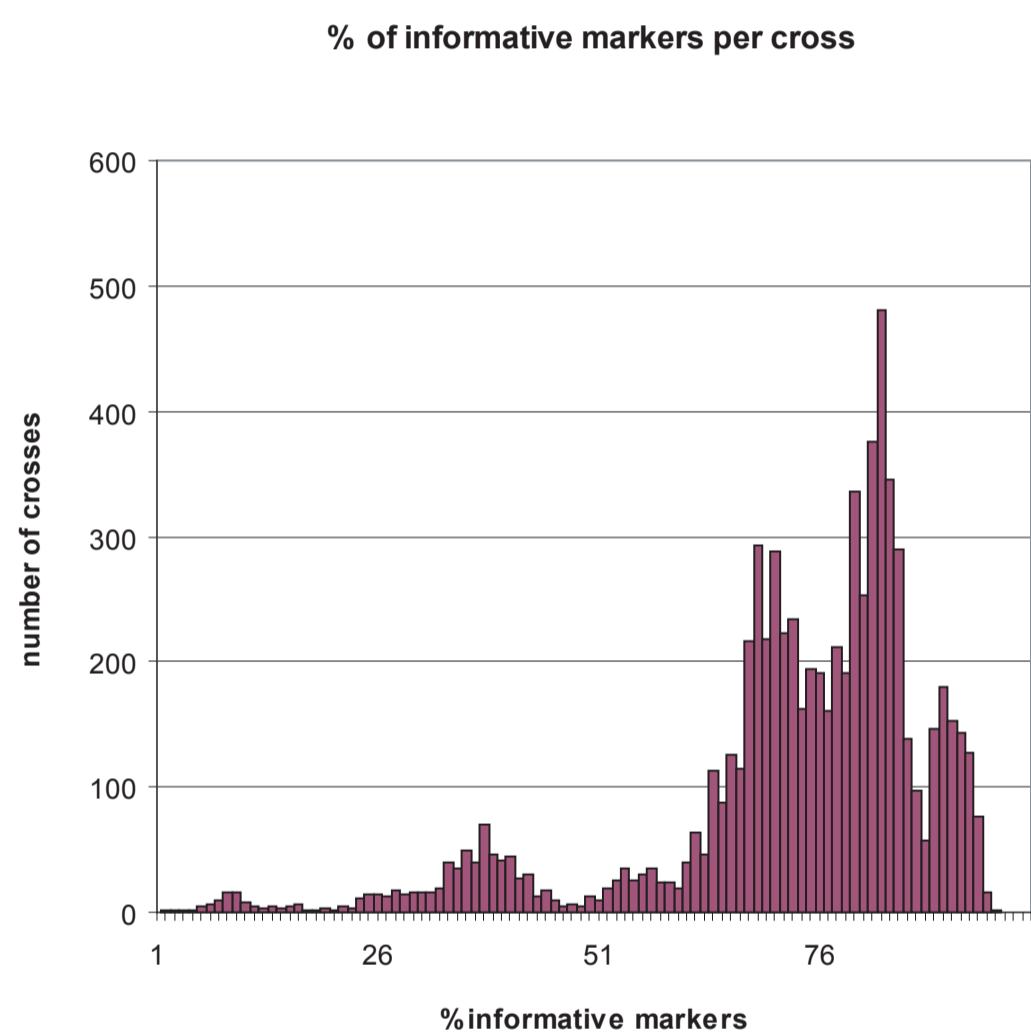

**Supplementary Figure 1**

Supplement: Additional File 3 — supplementary Figure 1The number of informative markers per cross. Pairwise combinations of 122 inbred rat strains were analyzed to determine the number of markers that were polymorphic for each cross. The number of informative crosses was plotted against the number of informative markers for each pair of strains. The tail on the left side of the histogram displays crosses between closely related substrains, including a cluster of ACI, BN, F344, and SHR strains. The mean number of informative markers per cross for all pairs of strains analyzed was 259 ± 21 SD (72.5% of 357 markers). [file 1471-2156-7-19-S3.pdf]
